# Correction: Knowledge, attitude, perception, and preventative practices towards COVID-19 in sub-Saharan Africa: A scoping review

**DOI:** 10.1371/journal.pone.0253833

**Published:** 2021-06-22

**Authors:** The PLOS ONE Staff

The second author’s name is spelled incorrectly. The correct name is: Emmanuella Chinonso Osuala. The correct citation is: Nwagbara UI, Osuala EC, Chireshe R, Bolarinwa OA, Saeed BQ, Khuzwayo N, et al. (2021) Knowledge, attitude, perception, and preventative practices towards COVID-19 in sub-Saharan Africa: A scoping review. PLoS ONE 16(4): e0249853. https://doi.org/10.1371/journal.pone.0249853

The publisher apologizes for the errors.

## References

[1] Nwagbara UI, Osual EC, Chireshe R, Bolarinwa OA, Saeed BQ, Khuzwayo N, et al. (2021) Knowledge, attitude, perception, and preventative practices towards COVID-19 in sub-Saharan Africa: A scoping review. PLoS ONE 16(4): e0249853. 10.1371/journal.pone.0249853 33872330 PMC8055009

