# Supplementary material for: Knowledge, attitude, perception, and preventative practices towards COVID-19 in sub-Saharan Africa: A scoping review
Source: PLoS One. 2021 Apr 19;16(4):e0249853. doi: 10.1371/journal.pone.0249853 (PMC8055009; doi:10.1371/journal.pone.0249853)
Supplement: S2 Appendix — (DOCX) [file pone.0249853.s002.docx]

**S2 Appendix. PubMed Search strategy**

| **Keyword search** | **Date of search** | **Search engine** | **Number of publications retrieved** |
| --- | --- | --- | --- |
| ((((("knowledge"[MeSH Terms] OR "knowledge") AND ("attitude"[MeSH Terms] OR "attitude"[All Fields] OR "attitudes"[All Fields])) AND ("perception"[MeSH Terms] OR "perception"[All Fields] OR "perceptions"[All Fields])) AND ("practice")) AND ("severe acute respiratory syndrome coronavirus 2"[Supplementary Concept] OR "severe acute respiratory syndrome coronavirus 2"[All Fields] OR "ncov"[All Fields] OR "2019-nCoV"[All Fields] OR "COVID-19"[All Fields] OR "SARS-CoV-2"[All Fields] OR ((coronavirus[All Fields] OR "cov"[All Fields]))) OR ("coronavirus"[MeSH Terms] OR "coronavirus"[All Fields])) AND ("SubSaharan Africa"[ All Fields] OR "sub-Saharan"[All Fields] OR "Angola" [All Fields] OR "Benin" [All Fields] OR "Botswana" [All Fields] OR "Burkina Faso" [All Fields] OR "Burundi" [All Fields] OR "Cameroon" [All Fields] OR "Cape Verde" [All Fields] OR "Central African Republic" [All Fields] OR "Chad" [All Fields] OR "Comoros" [All Fields] OR "Congo" [All Fields] OR "Democratic Republic of Congo" [All Fields] OR "Cote d'Ivoire" [All Fields] OR "Djibouti" [All Fields] OR "Equatorial Guinea" [All Fields] OR "Eritrea" [All Fields] OR "Ethiopia" [All Fields] OR "Gabon" [All Fields] OR "Gambia" [All Fields] OR "Ghana" [All Fields] OR "Guinea" [All Fields] OR "Guinea‐Bissau" [All Fields] OR "Kenya" [All Fields] OR "Lesotho" [All Fields] OR "Liberia" [All Fields] OR "Madagascar" [All Fields] OR "Malawi" [All Fields] OR "Mali" [All Fields] OR "Mauritania" [All Fields] OR "Mauritius" [All Fields] OR "Mozambique" [All Fields] OR "Namibia" [All Fields] OR "Niger" [All Fields] OR "Nigeria" [All Fields] OR "Rwanda" [All Fields] OR "Sao Tome" [All Fields] OR "Senegal" [All Fields] OR "Seychelles" [All Fields] OR "Sierra Leone" [All Fields] OR "Somalia" [All Fields] OR "South Africa" [All Fields] OR "South Sudan" [All Fields] OR "Sudan" [All Fields] OR "Swaziland" [All Fields] OR "eSwatini" [All Fields] OR "Tanzania" [All Fields] OR "Togo" [All Fields] OR "Uganda" [All Fields] OR "Zambia" [All Fields] OR "Zimbabwe" [All Fields])) Filters: from 2019/12/1 - 2020/10/31 | 2020/09/25 | PubMed | 976 |
|  |  |  |  |
