# Supplementary material for: Knowledge, attitude, perception, and preventative practices towards COVID-19 in sub-Saharan Africa: A scoping review
Source: PLoS One. 2021 Apr 19;16(4):e0249853. doi: 10.1371/journal.pone.0249853 (PMC8055009; doi:10.1371/journal.pone.0249853)
Supplement: Attachment — Submitted filename: Comments .docx [file pone.0249853.s004.docx]

**Methodology**

-This is an interesting topic to study due to the nature of the subject matter as it is the subject in the mouth of everyone in the whole world today. The authors have clearly presented their work and in a good professional manner however they may consider these few remarks that could also add value to their great work which they have done.

-In exclusion criteria they may consider to explain the reason for excluding infants, we should not assume that every leader will know it.

-On discussion the authors, could run away from presetting the results again but rather explain their meaning only.

-On results presentation, if they could add some graphs from the results could also add value to the way the results are being presented, for example the graph could show total division of 28 scores on K A P.

-On Results and discussion, the researchers may also consider dividing the sub-Saharan countries in general into regions for regional comparisons to see which region does well and why its impacts on KAP.
