# Supplementary material for: Knowledge, attitude, perception, and preventative practices towards COVID-19 in sub-Saharan Africa: A scoping review
Source: PLoS One. 2021 Apr 19;16(4):e0249853. doi: 10.1371/journal.pone.0249853 (PMC8055009; doi:10.1371/journal.pone.0249853)
Supplement: Attachment — Submitted filename: Response to Reviewers.docx [file pone.0249853.s005.docx]

**Author’s response to the reviews**

**Title:** Knowledge, attitude, perception, and preventative practices towards COVID-19 in sub-Saharan Africa: a scoping review

**Authors:**

**Ugochinyere I. Nwagbara:** [216045259@stu.ukzn.ac.za / ugochinyereijeoma@gmail.com](mailto:216045259@stu.ukzn.ac.za%20/%20ugochinyereijeoma@gmail.com)

**Emmanuella Chinonso Osuala:** [218024179@stuukznac.onmicrosoft.com](mailto:218024179@stuukznac.onmicrosoft.com) / osualachinonso@yahoo.com

**Rumbidzai Chireshe:** [216041773@stu.ukzn.ac.za](mailto:216041773@stu.ukzn.ac.za) / rchireshe1@yahoo.ca

**Obasanjo Afolabi Bolarinwa:** [219098880@stu.ukzn.ac.za](mailto:219098880@stu.ukzn.ac.za) **/** bolarinwaobasanjo@gmail.com

**Balsam Qubais Saeed:** bsaeed@sharjah.ac.ae

**Nelisiwe Khuzwayo:** Khuzwayone@ukzn.ac.za

**Khumbulani W. Hlongwana:** [Hlongwanak@ukzn.ac.za](mailto:Hlongwanak@ukzn.ac.za)

Manuscript PONE-D-21-04794

We are very grateful for the reviews provided by the editor and the reviewers of this manuscript. The comments were very useful. We have revised the manuscript according to the reviewer’s comments. Below is the point-by-point response to the reviewers’ comments. Please find attached a revised version of the manuscript with tracked changes highlighted in ‘red’.

**Responses to reviewers’ queries/ comments**

**Reviewer Comments**

This is an interesting topic to study due to the nature of the subject matter as it is the subject in the mouth of everyone in the whole world today. The authors have clearly presented their work and in a good professional manner however they may consider these few remarks that could also add value to their great work which they have done.

**Authors’ Response**

We appreciate the compliment, thank you.

-In exclusion criteria they may consider to explain the reason for excluding infants, we should not assume that every leader will know it.

**Authors’ Response**

Thanks, we have included the reason for excluding infants (page 7, lines 151-152).

-On discussion the authors, could run away from presetting the results again but rather explain their meaning only.

**Authors’ Response**

The authors have rearranged the discussion section and removed the repeated results (page 30-32).

-On results presentation, if they could add some graphs from the results could also add value to the way the results are being presented, for example the graph could show total division of 28 scores on K A P.

**Authors’ Response**

The authors have added a graph – Fig 3 (page 26, lines 3-5) on the results to demonstrate the KAP components of the included studies and elaborated on it (page 11, lines 264-270).

-On Results and discussion, the researchers may also consider dividing the sub-Saharan countries in general into regions for regional comparisons to see which region does well and why on KAP.

**Authors’ Response**

Thanks for this great suggestion, the authors have divided the SSA countries into regions on the results (page 11, lines 270-276), and on the discussion (page 31, lines 114-122).
